# Supplementary material for: Enhancing IDS for the IoMT based on advanced features selection and deep learning methods to increase the model trustworthiness
Source: PLoS One. 2025 Jul 2;20(7):e0327137. doi: 10.1371/journal.pone.0327137 (PMC12221068; doi:10.1371/journal.pone.0327137)
Supplement: Attachment — Submitted filename: Response to Reviewers.docx [file pone.0327137.s002.docx]

Dear Professors.

We would like to thank the editorial board and reviewers for their insightful comments on the paper. Your feedback greatly improved the paper's clarity and research depth. Below are responses to each comment received, indicating the points of revision that are modified according to your values recommendations. We hope the amended work will meet scientific expectations.

**Comments of the Esteemed Reviewer 1 and Our Responses:**

1. **Organization of the article is poor.**

*Response:* We appreciate this feedback which is improving our research.

We have reorganized the article structure from abstract to references and focused on the Methodology section to make it more streamlined and clear.

2. **The figures are not embedded in the text which affects the readability of the text.**

*Response:* We thank the reviewer for his valuable feedback

Following the journal's guidelines, we attached the figures independently and not within the text. However, we have taken care to indicate the locations of appropriate figures within the text to enhance understanding of the text.

We welcome any additional comments to improve the paper.

3. **Even though the title says IoMT, all datasets are not IoMT datasets.**

*Response:* We appreciate your feedback on the depth of the result analysis.

**CICIDS2017** is a comprehensive network dataset that encompasses attack patterns, such as DDoS and Man-in-the-Middle, commonly found in IoMT scenarios.

**WUSTL-EHMS-2020** is specific to the Internet of Medical Things, encompassing biometric and network data from medical devices. This makes its combined use optimal for assessing general and healthcare-related threats.

To clarify this, we have explicitly explained its relevance in the dataset description section.

4. **Many abbreviations are used which are not expanded during their first use.**

*Response:* We have carefully reviewed and ensured that all abbreviations are expanded upon their first mention.

5. **Methodology is not clear. Since the paper uses advanced feature selection process, comparison has to be done with reference to the features selected and the importance of these feature in the real life context.**

*Response:* We appreciate your feedback regarding the clarity of the methodology.

In the revised manuscript, we have provided a detailed explanation of the methodology, especially the advanced feature selection process. Figure 1 has been included to visually illustrate the steps involved, helping to clarify the flow and rationale behind our approach. Where the suggested changes have been incorporated in the Proposed Model section after the title of Figure 1. And the following are our justifications:

Figure 1 after updated.

**Clarification of Feature Selection Process:**

The methodology follows a structured process that begins with selecting relevant features from the raw data, followed by applying Information Gain (IG) and Recursive Feature Elimination (RFE) as feature selection techniques.

**The IG method** was chosen for its ability to quantify the relevance of individual features by measuring their contribution to the target variable. This helps in identifying which features are most useful for classification tasks. On the other hand.

**The RFE** was employed to iteratively eliminate less important features, ensuring that only the most relevant ones remain for training the model. This combination of methods ensures that we not only select the most informative features but also reduce potential overfitting by eliminating redundant or irrelevant features.

IG and RFE were selected due to their ease of implementation and efficacy in managing the intricate datasets utilised in the study (e.g., CICIDS2017 and WUSTL-EHMS-2020), while maintaining a balance between correctness, complexity, and minimising computational expenses. These approaches maintain the interpretability of the original features and facilitate real-time applications in IoMT contexts.

The selection of the top 50% of features was based on empirical validation, optimizing both model performance and computing efficiency. This method preserved elevated accuracy while diminishing complexity, which is essential for IoMT applications. Cross-validation validated optimal performance with this ratio, reducing overfitting risks and ensuring robustness.

We have already added more clarification to the Proposed Model section and modified Figure1 according to your comments.

6. **Result Analysis is also not satisfactory.**

We sincerely appreciate the reviewer’s valuable feedback.

*Response:* We have provided comparisons with state-of-the-art studies, as shown in Tables 8 and 9, and included detailed performance evaluations using multiple metrics such as accuracy, Precision, recall, f1 score, detection rate, error rate, and memory consumption, as shown in Tables 6, 7, 10, and 11. We also added confusion matrices, Figures 8 and 9, and performance charts, Figures 14 and 15, in support of such depth in analysis. We acknowledge that we must strengthen the interpretation of our results, and for this.

We enriched the discussion better to bring out the practical benefits of the proposed model.

7. **Lot of grammatical errors are there. Thorough proofreading has to be done.**

*Response:* Comprehensive proofreading has been conducted to improve grammar and readability.

**Comments of the Esteemed Reviewer 2 and Our Responses:**

1. **The abstract is well-written, and the topic is interesting. Provide a brief mention of numerical improvements (e.g., percentage increase in accuracy or F1 score).**

*Response:* We appreciate your suggestion**.**

The proposed model achieves 99.61% accuracy with (CICIDS2017) and 99.93% accuracy with (WUSTL-EHMS-2020), surpassing state-of-the-art methods by 2.1% and 1.5%, respectively, while reducing training time as shown in Table 7 with WUSTL-EHMS-2020.

we added these details to the Abstract according to your suggestion.

2. **Why were IG and RFE chosen over other feature selection methods? A brief rationale could strengthen the argument.**

*Response:* We highly appreciate your deep feedback

IG and RFE have been selected because they are relatively simple to apply, and efficient in processing complex datasets that will be used here, such as CICIDS2017 and WUSTL-EHMS-2020, providing the best balance among accuracy, complexity, and reduction of computational costs. In addition, the approaches preserve the interpretability of the original features and enable their straightforward application within the IoMT contexts.

We have already added more clarifications to the Proposed Model section according to your comment.

3. **The use of Deep Autoencoder for dimensionality reduction is valid, but its specific advantage in this context needs clearer articulation.**

*Response:* We appreciate your deep feedback

DAE was chosen for its ability to learn nonlinear representations of high-dimensional data while preserving critical information, which reduces noise and improves model efficiency in an IoMT environment, where the data is feature-rich (e.g., biometric and network data), DAE helps reduce dimensionality without losing essential features, making the model lighter and faster to train and execute and increases the model’s focus on the patterns most relevant for classification. This effectiveness has been demonstrated experimentally by reduced memory usage (Tables 9 and 10, Figures 16 and 17) and enhanced performance in attack detection.

We highlighted that in the DAE section after Fig.4.

4. **Clarify how the combination of IG, RFE, and Deep Autoencoder improves upon prior works.**

*Response:* We acknowledge the importance of your feedback.

RFE, IG, and DAE were chosen due to their effectiveness in dealing with security challenges in IoMT environments. IDS suffers from high data dimensions and the need to achieve accurate threat detection with record response time. IG was adopted as a fast and effective filtering method to identify the most influential features without increasing the computational load, while RFE works to reduce overfitting by removing unnecessary features iteratively. As for DAE, it was adopted as an effective tool that showed a high ability to reduce dimensions while preserving the important information of the features, outperforming other methods, which was positively reflected in enhancing the accuracy of the model. Based on the tests we conducted while applying the proposed model on two datasets (CICIDS2017 and WUSTL-EHMS-2020), it outperformed in achieving 99.61% accuracy with CICIDS2017 and 99.93% with WUSTL-EHMS-2020, thus surpassing modern methods in terms of accuracy, detection rate, and reduced training time. Therefore, the selection of IG, RFE, and DAE can be considered as the optimal choice for computational accuracy, performance speed, and effective threat detection in IoMT systems.

5. **Highlight why selecting the top 50% of features is optimal.**

*Response:* We evaluate your important feedback

The selection of the top 50% of features was based on empirical validation, optimizing both model performance and computing efficiency. This method preserved elevated accuracy while diminishing complexity, which is essential for IoMT applications. Cross-validation validated optimal performance with this ratio, reducing overfitting risks and ensuring robustness.

6. **While the datasets are mentioned, their relevance to IoMT and the specific challenges they address are not fully explained. Why are these datasets suitable for evaluating IoMT IDS?**

CICIDS2017: Encompasses several network attacks (e.g., DDoS and Botnet), assessing the model's capacity to generalize to typical IoT settings.

WUSTL-EHMS-2020: focuses on healthcare-related assaults (e.g., data injection and identity theft), highlighting distinct IoMT concerns such as the sensitivity of medical data and the diversity of devices.

The combination of both datasets guarantees a thorough assessment of the model in practical situations.

7. **IDS and IoMT are defined again and again while they should be abbreviated at 1 time only when it is first used.**

*Response:* Thank you so much for your feedback

We have reviewed and ensured that each abbreviation is defined only once.

8. **The section on Deep Neural Networks (DNNs) is generic and lacks details specific to the study, such as the architecture, hyperparameters, or training strategies used.**

*Response:* We would like to thank you for suggesting that the explanation of the DNN section.

The DNN model in this study is designed to fulfill the requirements of IDS in IoMT, considering the problems of high dimensions and complexity of data. Three hidden layers of 64, 32, and 16 neurons using ReLU activation handle non-linear input. This number of neurons was chosen to reduce computational complexity and extract key information. The last layer consists of one neuron with the Sigmoid activation function for binary classification (BENIGN or ATTACK). The learning rate was set to 0.001 with the Adam optimizer, and batch size was 256, both carefully tuned in order to balance performance and complexity. Early Stopping and Reduce Learning Rate on Plateau enhanced model stability and reduced overlearning. This architecture provides perfect accuracy for two datasets.

We have added more details to the DNN section and explained how the model and its components are designed, the section has become more detailed and comprehensive.

9. **Discuss the scalability of these methods and their potential deployment in practical IoMT applications. Mention challenges such as data privacy, device compatibility, or computational constraints in real-time scenarios.**

*Response:* We appreciate the reviewer’s valuable feedback and insightful comments,

The proposed model is scalable and compatible with IoMT computational challenges, so many connected medical devices (e.g. wearable sensors) can be added using best feature selection techniques (IG, RFE) and dimensionality reduction techniques (DAE), which reduce computational limitations. Scalability and working with resource-constrained devices are possible due to the low memory utilization (Figures 16 and 17) and execution time (3.2 seconds).

Challenges that the model may face are: It may need to be fine-tuned when applied to another dataset because it relies on two standard datasets for training, compatibility with different devices, and privacy risks. Therefore, future work will focus on adaptive learning techniques and hybrid models to address and solve these problems.

We have already added more clarifications to the sections according to your comment.

10. **Highlight why the TFS-U subset outperformed other subsets. Reflect on why subsets like TFSIG or TFSRFE underperformed relative to TFS-U.**

*Response:* We are grateful for this valuable comment,

The reasons for the superiority of the TFS-U (Union of Top Feature Subsets) subset is the combination of more comprehensive features as it contains a wider and more diverse set of features that reflect different aspects of the data, which is positively reflected in the model's ability to distinguish between natural and attack patterns. Reducing information loss as a subset such as TFS-I (intersection) may exclude some important features that are not present in both intersecting subsets (TFSIG, TFSRFE) such as features related to uncommon attacks, while TFS-U maintains the diversity of features. TFSIG and TFSRFE suffer from limitations such as an apparent bias towards individual selection criteria especially in dynamic IoMT environments, which may lead to neglecting features with an indirect effect.

The experimental results and comparison of the model with recent studies showed the clear superiority of the model with the TFS-U subset, as appeared in tables 6,7,8,9.

We highlighted that in the Results and Measurements section after Table 7.

11. **Emphasize the novelty and advantages of combining DAE and DNN with selected features.**

*Response:* We are grateful for this valuable comment,

Based on practical experience and some previous studies, it has been proven that the combination of DAE and DNN with the selected features has a positive impact on the design of IDS in IoMT environments, as the proposed approach demonstrated accuracy in classification and efficiency in work, as it clearly shows superior results, reduced computational complexity, reduced memory consumption, reduced error rates, accelerated training time, stability and overcoming overfitting, as well as the possibility of expansion and protection of sensitive data because reducing dimensions via DAE reduces the risk of accessing all private data, which reduces the risk of hacking.

I added Emphasize accounting to your valuable comment in the Proposed Model section after Fig. 5.

12. **While the model is stated to be suitable for real-time threat detection, there is no mention of potential latency or resource constraints.**

*Response:* We thank you for your valuable comments that helped improve the quality of the paper.

The proposed model relied on advanced feature selection techniques (IG and RFE) to focus on the most useful features and leave the others. The model also benefited from DAE to reduce the dimensions of the selected features, which was reflected in significantly improving efficiency and accuracy, as the model achieved an accuracy of 99.61% with CICIDS2017 and 99.93% with WUSTL-EHMS-2020. Also, choosing the appropriate techniques for the model led to reducing the training time to 3.2 seconds. Memory consumption was also significantly reduced, which may make it compatible with devices with limited resources. The model also outperforms metrics such as AUC and MCC compared to recent methods, which indicates its robustness and effectiveness. These capabilities position the approach as an important step toward enhancing the security of IoMT networks. Future work will focus on calculating and improving latency to meet the requirements of critical applications and ensure compatibility with time-sensitive IoMT environments.

13. **Although the model is said to reduce computational complexity, no quantitative evidence is provided in the conclusion.**

*Response:* We appreciate the reviewer for this great comment.

We fully concur with your observation regarding the necessity of presenting quantifiable proof to substantiate statements of the reduction of computing complexity. The study presented quantifiable results demonstrating the model's efficacy in diminishing computing complexity, described as follows:

I. Reducing training duration: As evidenced in Table 7, the suggested model markedly diminished the training duration utilizing the WUSTL-EHMS-2020 dataset, with the training time decreasing from 12.84 seconds (with original features) to only 3.2 seconds when implementing the proposed model (for the TFS-U subset).

II. Reducing memory consumption: Feature selection (IG, RFE) and dimensionality reduction techniques (DAE) led to a significant reduction in memory consumption as shown in Tables (10 and 11), wherewith the CICIDS2017 dataset the memory consumption reduced from 2.88 GB (for the full set) to 1.16 GB, as well as the WUSTL-EHMS-2020 dataset, reduced from 12.96 MB to 4.11 MB (for the TFS-U subset).

III. Enhancing performance: The model decreased the number of input features to 50%, which substantially lowered computational complexity while preserving high classification accuracy, achieving 99.61% accuracy with CICIDS2017 data and 99.93% with WUSTL-EHMS-2020 data. This reduction facilitated the acceleration of the training and prediction processes.

We appreciate your critical comments and have incorporated a clarification in the Conclusion based on your insightful opinion to enhance the overall understanding.

14. **Specific directions for improvement, such as integrating more diverse datasets, optimizing the model for resource-constrained environments, or addressing new threat types, are missing.**

*Response:* We appreciate the reviewer's observation about the need for specific directions for improvement. In future work, the model will be tested on more diverse datasets to cover more potential scenarios in IoMT environments, the model will be improved for environments with limited resources, new and evolving types of threats will be addressed, and latency will be calculated and reduced. These suggestions will contribute to enhancing the model's ability to meet the increasing security challenges in IoMT environments.

We have included these directions in the conclusion section to illustrate paths for improvement of the model.

We appreciate your valuable comments and thank you for your constructive review. We look forward to benefiting from your great experience in the future.
