# Supplementary material for: Enhancing IDS for the IoMT based on advanced features selection and deep learning methods to increase the model trustworthiness
Source: PLoS One. 2025 Jul 2;20(7):e0327137. doi: 10.1371/journal.pone.0327137 (PMC12221068; doi:10.1371/journal.pone.0327137)
Supplement: Attachment — Submitted filename: Response_to_Reviewers_auresp_2.docx [file pone.0327137.s003.docx]

**Dear Editor and Reviewers,**

We sincerely thank you for your thorough and insightful feedback, which has significantly helped us improve the quality and clarity of our manuscript. We have carefully addressed each comment by incorporating additional details, restructuring sections, and providing deeper justifications to enhance the scientific rigor and practical relevance of our work. Below, we outline our responses to each comment, detailing the revisions made to the manuscript, including new paragraphs, tables, and analyses, to ensure alignment with your recommendations. We believe these changes strengthen the manuscript and better highlight its contributions to IoMT cybersecurity.

1. **The authors state that they have reorganized the article.
   Issue: There is no explicit mention of how the structure was improved. The response is vague and lacks specific details on how readability, logical flow, or coherence has been enhanced.**

**ANS:**

We thank the reviewer for their valuable comments. We have reorganized the manuscript to improve the logical flow and coherence of the content by providing a clear roadmap for readers. To enhance clarity, the following paragraph has been added to the end of the introduction section to guide readers through the research structure:

"The remainder of this paper is structured as follows: Section 2 is a comprehensive review of related work. Section 3 describes the proposed IDS model framework with data preprocessing, feature selection, dimensionality reduction, and classification. Section 4 provides an overview of the experimental setup and datasets. Section 5 reports and discusses the evaluation results. Section 6 compares the proposed model with state-of-the-art approaches. Section 7 concludes the study. Finally, Section 8 provides future directions."

2. **The authors justify CICIDS2017 by stating that it contains attacks relevant to IoMT environments.
   Issue: CICIDS2017 is still a general IoT dataset, not an IoMT-specific one. Their justification does not fully address why a non-IoMT dataset is suitable.**

**ANS:**

We thank the reviewers for their insightful comments.

We fully agree with the reviewer that the CICIDS2017 dataset is not exclusively rel for IoMT.

Therefore, WUSTL-EHMS-2020 was used as the primary reference because it represents a real IoMT environment.

CICIDS2017 was used as a supplementary dataset because it includes attacks relevant to IoMT environments.

Notably that several recent studies in the field of IoMT have also utilized the CICIDS2017 dataset as a reference for evaluating intrusion detection systems, given its variety of attacks and relevance to the behavior of IoMT.

For example, (Manimurugan et al.,2020) and (Balhareth & Ilyas, 2024) used CICIDS2017 to evaluate the performance of IDS in the IoMT context.

The following paragraph has been added to the end of the (Dataset Overview) section to explain the reason behind selecting each dataset and to reference studies that have used CICIDS2017 for the same reason:

" This study uses WUSTL-EHMS-2020, which includes biometric and network data from medical devices, for IoMT. CICIDS2017, a comprehensive dataset that encompasses attacks across various IoT scenarios, was utilized to broaden the evaluation. By integrating the two datasets, the model can generalize across contexts and test the system's ability to respond to complex and diverse challenges. Recent research like [36] and [37] used the CICIDS2017 dataset to evaluate IoMT security systems, supporting this methodology."

3. **They reviewed and expanded abbreviations at first mention.
   Issue: This needs verification. If certain abbreviations remain undefined upon first usage, they need to correct this systematically.**

**ANS:**

We thank the reviewer for their valuable comments. All abbreviations are defined in Table 1.

Table 1: List of Abbreviations and Symbols.

| **Abbreviation** | **Full Definition** | **Abbreviation** | **Full Form/Definition** |
| --- | --- | --- | --- |
| IoT | Internet of Things | IoMT | Internet of Medical Things |
| IDS | Intrusion Detection System | IG | Information Gain |
| RFE | Recursive Feature Elimination | DAE | Deep Autoencoder |
| DNN | Deep Neural Network | WUSTL-EHMS-2020 | Washington University in St. Louis Electronic Health Monitoring System 2020 Dataset |
| ML | Machine Learning | CICIDS2017 | Canadian Institute for Cybersecurity Intrusion Detection System 2017 Dataset |
| DL | Deep Learning | ACC | Accuracy |
| PRE | Precision | DR | Detection Rate |
| FNR | False Negative Rate | FPR/FAR | False Positive Rate / False Alarm Rate |
| ICI | Incorrect Classification Instances | ER | Error Rate |
| MCC | Matthew’s Correlation Coefficient | AUC | Area Under the Curve |
| TP | True Positive | TN | True Negative |
| FP | False Positive | FN | False Negative |
| ReLU | Rectified Linear Unit (activation function) | TFSIG | Top Fifty Percent-Feature Subset based on Information Gain |
| TFS-U | Union of TFSIG and TFSRFE | TFSRFE | Top Fifty Percent-Feature Subset based on Recursive Feature Elimination |
| TFS-I | Intersection of TFSIG and TFSRFE | HIPAA | Health Insurance Portability and Accountability Act (US data privacy regulation) |
| DoS | Denial of Service | GDPR | General Data Protection Regulation (EU data privacy regulation) |
| DDoS | Distributed Denial of Service | MitM | Man-in-the-Middle Attack |
| PSO | Particle Swarm Optimization |  |  |

4. **They added details on IG and RFE selection, as well as the impact of feature selection.
   Issue: The response focuses on theoretical justification but does not compare the selected features with alternative feature selection methods or explain the practical significance of these features in a real IoMT deployment. An explicit comparison with alternative selection methods and a deeper justification of why the chosen features matter in an IoMT-specific security context is needed to be included.**

**ANS:**

(The following paragraph was added to the "Features Selection and Reduction Approach" section)

Thank you for this insightful comment. We agree that theoretical justifications alone are insufficient, so we will provide a more in-depth comparison between different methods and select the most relevant features for the medical environment, as shown below:

" Recent studies show that feature selection strategies improve IDS efficiency and accuracy in IoT contexts. Effective model complexity, accuracy, and training time reduction are essential for resource-constrained IoT systems that require fast and dependable responses. Similarly, the study [27], combining RFE-RIDGE with only 13 important features, improved classification performance to 97.85% accuracy and 0.03 false alarm rate. Reduced data dimensionality enhanced computational performance and complexity. The study [28] suggested IGRF-RFE, a hybrid technique for choosing key features. Selecting 23 of 42 UNSW-NB15 characteristics boosted classification accuracy to 84.24%. These approaches have increased computing efficiency and reduced complexity while preserving IDS performance. In [8], the authors presented LRGU-MIFS feature selection. This method calculates feature recurrence, decreasing feature duplication. Only 30 dataset features were picked using this method. This method improves performance and computational complexity, achieving 95.5% classification accuracy in IoMT.

Considering these results, the innovative approach to this work, which combines IG and RFE, using intersection and union methods, and DAE for dimensionality reduction, is effective and applicable in IoMT contexts. It balances high performance and computational efficiency while lowering feature size to the smallest set without compromising model accuracy. These features, including biometric and network flow data, are critical for detecting IoMT-specific threats such as spoofing or data injection"

5. **They expanded on the discussion, added confusion matrices, and included additional tables.
   Issue: There is still insufficient discussion on practical implications, statistical significance of improvements, and how these findings translate into real-world benefits for IoMT networks. The authors should provide deeper insights into what these results mean in practical IoMT deployments—how do they handle adversarial attacks, real-time performance in constrained environments, and device heterogeneity?**

**ANS:**

We sincerely thank the reviewer for this valuable and constructive feedback. Based on this valuable feedback, we have made the following modifications to the manuscript to clarify the practical aspects further:

i. Statistical significance of improvements:

(The following paragraph was added to the "Experimental Results and Analysis" section after Table 7.)

To assess the statistical reliability of the proposed model, ten independent experiments were conducted using the WUSTL-EHMS-2020 dataset. The proposed model achieved 99.823% average accuracy, a standard deviation of ±0.161, and a 95% confidence interval of ranging accuracy from 99.47% to 99.96%. As well as a one-sample t-test [40] against the baseline value of 99.5% returned a p-value of 0.00014, proving a statistically significant improvement is not because random variation.

Though the same models in other literature often have one performance measure, uniform performance across runs attests to the robustness of the proposed model.

ii. Real-World Practical Implications, Device Heterogeneity and Adversarial Attacks: Based on this valuable revision, we expand the Discussion section to show how our results assist IoMT networks:

(The following paragraph was added to the end of the "Experimental Results and Analysis" section after Fig.13)

the Proposed Model 99.9% and 99.6% accuracy at WUSTL-EHMS-2020 and CICIDS2017, respectively, indicate great promise of early cyberattack detection before they affect the connected medical devices. Remote patient monitoring systems can prevent service outages and data compromise by immediately detecting MitM or DDoS.

A light model and some carefully selected features reduce resource usage to render the model deployable on low-capacity devices like wearables. The model was trained and tested on heterogeneous, biometric signatures and network flows to capture the variability of IoMT device data and prove its versatility to varying operating environments.

iii. Real-Time Performance:

We have reformulated part of the conclusion to illustrate the model's suitability for IoMT environments with performance and resource constraints as follows:

"The model achieved low misclassification and error rates with a significant reduction in memory consumption and training time, making it suitable for real-time use on limited IoMT devices. Its lightweight architecture and efficient feature selection make it highly suitable for embedded deployment in wearable or mobile medical devices."

6. **They mentioned improvements in execution time and memory efficiency.
   Issue: The response lacks a detailed latency analysis for real-time scenarios and does not quantify computational overhead. The authors should at least include numerical latency measurements, resource utilization data, and a discussion of the challenges in deploying the model on low-power IoMT devices.**

**ANS:**

We thank the reviewer for his constructive feedback. We fully agree on the importance of including an analysis of latency and computational overhead, especially when targeting real-time applications and resource-constrained devices in an IoMT environment.

In this research, we focused on improving performance by reducing model complexity using feature selection techniques (IG and RFE) and dimensionality reduction using DAE, which resulted in significant memory consumption reductions and reduced training time, as shown in Tables 6, 7, 10, and 11.

However, we did not conduct direct measurements of response time or resource consumption at the hardware level, and we acknowledge that this is a limitation of the current work.

Our main goal was to reduce model complexity using feature selection techniques (IG and RFE) and dimensionality reduction using DAE.

These improvements indicate a suitable possibility for deployment on lightweight and resource-constrained devices.

i. In response to your valuable comments, we have enhanced the Discussion section by adding an explanation of the challenges of deployment on low-power IoMT devices, as follows:

"The proposed intrusion detection system model enhanced accuracy, memory consumption, and training time, but real-time latency and hardware-specific performance were not explicitly measured. The DNN architecture used in this study is relatively simple, using a small number of layers and nodes, and dimensionality reduction using DAE reduced the computational burden.

However, implementation on low-power devices such as Raspberry Pi, microcontrollers presents processing, memory, and power consumption management issues. The approach has endpoint implementation potential, however, latency, CPU utilization, and power consumption must be tested. Future focus will be on these limitations."

ii. A paragraph has also been added in the Future Work section confirming our intention to conduct a comprehensive evaluation of response time and computing load on real platforms to ensure the model is ready for practical deployment.

"The proposed model has shown superior performance, but to ensure sustainable performance in resource-constrained and critical IoMT environments, it can be integrated with more comprehensive datasets to cover a wider range of uncommon or advanced attacks scenarios, addressed by adaptive, hybrid learning, and reinforcement learning strategies, and evaluated for latency, computational, and feasibility. Experimental work with Raspberry Pi or ESP32 to better understand the approach's practical uses in time-sensitive medical applications."

7. **They provided reduced training time and memory consumption statistics.
   Issue: The provided numbers do not compare their approach with alternative methods or justify trade-offs. Reducing complexity while retaining model effectiveness in real-time applications is critical. Comparisons with other lightweight IDS models and a justification for why this specific trade-off is optimal are needed.**

**ANS:**

We thank the reviewer for this insightful comment. In response to this comment, we have revised the manuscript to include explicit comparisons with other lightweight intrusion detection models, both in terms of training time and memory consumption, as shown in Tables 8–10 and Figures 16–17.

In addition, we have added a new paragraph to the Discussion section to explain the rationale for the balance between reducing complexity and maintaining performance in the proposed model design.

Our reliance on a combination of feature selection techniques (IG and RFE) and dimensionality reduction using DAE allows for a highly compact representation without losing accuracy—and even achieving higher performance. This balance makes the model well-suited for IoMT environments.

Below are the modifications we made to the paper in response to your valuable comments.

| Memory Consumption | WUSTL-EHMS-2020 | CICIDS2017 |
| --- | --- | --- |
| Original Features | 12.96 MB | 2.88 GB |
| TFSIG | 2.74 MB | 0.84 GB |
| TFSRFE | 2.74 MB | 0.84 GB |
| TFS-U (Union) | 4.11 MB | 1.16 GB |
| TFS-I (Intersection) | 1.37 MB | 0.53 GB |

**Table 10.** Memory Consumption Across Feature Sets in WUSTL-EHMS-2020 and CICIDS2017.

"Unlike conventional IDS models that demand extensive computation, the proposed IDS for IoMT in this paper significantly reduces computational complexity while maintaining high accuracy, 99.93% and 99.61% for WUSTL-EHMS-2020, and CICIDS2017 respectively, as shown in Tables 6 and 7. Using the proposed IDS, memory consumption decreases from 12.96 MB to 4.11 MB for WUSTL-EHMS-2020 and from 2.88 GB to 1.16 GB for CICIDS2017, with training times reduced from 12.84 to 3.2 seconds for WUSTL-EHMS-2020 as shown in Table 6. The model’s low memory footprint and Low training time enable threat detection faster on resource-constrained IoMT devices [6]. This balance ensures reliable detection with minimal false negatives, while DAE enhances data privacy [37].

Compared to existing lightweight IDS like XMeDNN [25] and Ridge-CNN [24], proposed IDS exhibits superior accuracy while operating with reduced complexity. The proposed model has been carefully calibrated to maintain superior detection performance while achieving model consistency. "

8. **They added general directions for future research.
   Issue: The proposed future work remains superficial. It lacks a concrete roadmap addressing:
   How their model can be optimized for new IoMT attack types.
   How they plan to validate performance in real IoMT environments.
   Potential integration with real-world medical devices and compliance with IoMT security standards (e.g., HIPAA, GDPR).**

**ANS:**

We thank the reviewer for his valuable comments on the importance of deepening the discussion of future work. We fully agree with the respected reviewer that a clear and specific plan is needed to improve the current model and ensure its practical applicability in IoMT environments. In response to this comment, we have updated the "Future Work" section to address the points raised, and have added the following future work:

"It can be integrated with more comprehensive datasets to cover a wider range of uncommon or advanced attacks scenarios, addressed by adaptive, hybrid learning, and reinforcement learning strategies, and evaluated for latency, computational, and feasibility. Experimental work with Raspberry Pi or ESP32 to better understand the approach's practical uses in time-sensitive medical applications.

Additionally, explore and integrate real medical devices while complying with IoMT security and privacy requirements like HIPAA (US) and GDPR (EU). This will be achieved by applying advanced security technologies such as strong encryption, identity-based access management, and federated learning to ensure compliance with data privacy and processing laws."

We are deeply grateful for the constructive feedback provided by the reviewers, which has guided us in refining our manuscript to meet the highest standards of academic rigor and practical applicability. We hope that the revised manuscript meets your expectations. We remain open to any further suggestions to ensure the quality of the final publication.

Sincerely,
