# Supplementary material for: Enhancing IDS for the IoMT based on advanced features selection and deep learning methods to increase the model trustworthiness
Source: PLoS One. 2025 Jul 2;20(7):e0327137. doi: 10.1371/journal.pone.0327137 (PMC12221068; doi:10.1371/journal.pone.0327137)
Supplement: Attachment — Submitted filename: Response_to_Reviewers_auresp_3.docx [file pone.0327137.s004.docx]

**Dear Editor and Reviewers,**

We sincerely thank you for your thorough and insightful feedback, which has significantly helped us improve the quality and clarity of our manuscript. We have carefully addressed each comment by incorporating additional details, restructuring sections, and providing deeper justifications to enhance the scientific rigor and practical relevance of our work. Below, we outline our responses to each comment, detailing the revisions made to the manuscript, including new paragraphs, tables, and analyses, to ensure alignment with your recommendations. We believe these changes strengthen the manuscript and better highlight its contributions to IoMT cybersecurity.

1. **The abstract still includes high-level claims (e.g., “enhances detection efficiency”) but does not clearly describe the pipeline components (IG, RFE, DAE, DNN) or mention statistical significance tests.**

**ANS:**

Thank you for your constructive feedback. We have revised the abstract to explicitly describe the model pipeline and the statistical validation components. Specifically, we have included pipeline components and key statistical validation metrics, as below:

" The proposed model demonstrates superior performance in terms of accuracy, precision, recall, and F1 score. It achieves an accuracy of 99.93% on the WUSTL-EHMS-2020 dataset while reducing training time and attains 99.61% accuracy on the CICIDS2017 dataset. The model achieved an average accuracy of 99.82% ± 0.16% and a p-value of 0.0001 on the WUSTL-EHMS-2020 dataset, which refers to stable statistical improvement. This study shows that the proposed strategy decreases computational complexity and enhances IDS accuracy and efficiency in IoMT networks. "

This revision now ensures clarity and reflects the critical components of the methodology as you suggested.

2. **The response outlines prior works and justifies IG + RFE, but the architectural innovation and integration rationale remain vague.**

**ANS:**

Thank you for your valuable comments. We understand the need to clarify the rationale for innovation and architectural integration behind the proposed model. In response to your valuable suggestion, the discontinuity study has been included in the research after Table 8, as follows:

" As well as demonstrating how each model component affects performance, an ablation study was conducted to compare five model configurations:

Scenario 1: Processing raw data using DAE and DNN without feature selection (raw Features).

Scenario 2: Selecting the top 50% of features using IG-only with DAE and DNN (TFSIG).

Scenario 3: Retain the top 50% of features with DAE and DNN using RFE-only (TFSRFE).

Scenario 4: IG and RFE Integrating using the intersection of selected features with DAE and DNN (TFS-I).

Scenario 5: IG and RFE Integrating using the union of selected features with DAE and DNN (TFS-U).

As shown in Tables 7 and 8, the last scenario (TFS-U) showed a clear superiority in performance compared to the other scenarios. For example, accuracy increased to 99.93% using the WUSTL-EHMS-2020 dataset, compared to 90.196% using IG-only (TFSIG), and FAR decreased to 0.0007. This highlights the enhanced benefits of integrating these techniques into the model."

3. **The manuscript does not incorporate or mention any interpretability tool (e.g., SHAP, LIME) or provide qualitative analysis.**

**ANS:**

We sincerely thank the reviewer for highlighting the importance of interpretability in medical systems and for suggesting tools like SHAP, LIME, or Grad-CAM to enhance the trustworthiness model. We completely agree that interpretability is a critical aspect of deploying machine learning models in healthcare settings, where understanding the rationale behind predictions can significantly improve user confidence and adoption.

To address this concern, we have updated the "Future Direction" section of the manuscript to explicitly discuss the limitations of interpretability in the current work and propose the integration of advanced interpretability tools as part of future research.

4. **Authors acknowledge not performing hardware-specific latency analysis and only include memory and training time.**

**ANS:**

We thank the reviewer for pointing out the importance of analyzing latency and FLOPs as part of evaluating the computational performance of the model. Based on this observation, we conducted additional experiments to evaluate latency and FLOPs using the WUSTL-EHMS-2020 datasets. As shown in the following text, which was added to the research:

"Based on the study [41], which refers to the importance of inference latency and Floating Point Operations FLOPs, this work analyzed the approximate response time per sample (latency) and number of calculations (FLOPs) using the WUSTL-EHMS-2020 dataset. The model showed an inference latency of 112 ms for all features, decreasing significantly with feature selection: 70 ms (TFSIG), 68 ms (TFSRFE), 52 ms (TFS-I), and 59 ms (TFS-U). The total FLOPs for the model were estimated at 2,129 calculations.

According to the study [42], models achieving <100ms and <10,000 FLOPs are suitable for limited devices, and our model achieves 52–59ms and 2129 FLOPs, confirming its efficiency for deployment on Raspberry Pi and Jetson Nano."

5. **While the manuscript includes several foundational works on IoMT security and feature selection, it omits recent and relevant studies on lightweight intrusion detection, feature optimization strategies, and AI-based real-time threat analysis in IoT/IoMT environments. These omissions limit the depth and currency of the literature review.**

**ANS:**

We thank the reviewer for his important observation regarding the comprehensiveness and modernity of the references in the Related Work section. We fully agree with the importance of strengthening the literature review by incorporating recent studies relevant to IoMT. Based on the recommendation, the suggested studies have been included in the section as shown below:

"Recent literature, which focuses on innovative approaches to enhanced accuracy and operational efficiency, provides a deeper understanding of IDS in IoMT environments. For example, a study [20] presented an IDS model based on Vision Transformers, demonstrating how modern deep learning techniques can be used to efficiently process complex data. The research [21] focused on developing models capable of detecting zero-day attacks using machine learning techniques, reflecting the importance of generalization when dealing with unknown or unexpected data. Furthermore, the study [22] conducted a comprehensive review and detailed analysis of the challenges associated with using artificial intelligence to detect zero-day attacks, recommending the use of hybrid models and dimensionality reduction techniques to improve performance.

Finally, the study [23] demonstrated the potential for designing networks with attention-based architectures to achieve better integration of time-sensitive features, providing valuable insights applicable to highly sensitive IoMT environments. "

6. **The comparison focuses mostly on prior DNN-based models; non-DNN or transformer-based IDS are not addressed.**

**ANS:**

We thank the reviewer for his valuable comments on the importance of expanding the literature discussion to IDS models based on other techniques. This is discussed in the Related Works section, as shown below:

" The research [21] focused on developing models capable of detecting zero-day attacks using machine learning techniques, reflecting the importance of generalization when dealing with unknown or unexpected data. "

7. **Figures have been included, but clarity and resolution are still suboptimal. ROC curves and ablation tables are hard to read.**

**ANS:**

We thank the reviewer for their valuable comments on the clarity of the illustrations and the quality of the presentation. We recognize that the quality of the images plays a critical role in accurately communicating research results. Based on the recommendation:

The graphs (ROC curves and confusion matrices) have been redesigned with higher resolution, with improved clarity of the axes and legends, and axis labels.

The colors and display styles have been standardized across all graphs for consistency and ease of understanding.

In addition, Tables 7 and 8 have been updated to summarize the model performance results based on different feature sets (IG, RFE, intersection, and union).

8. **There are redundant claims (e.g., high accuracy, low memory) across the abstract, results, and conclusion.**

**ANS:**

We thank the reviewer for his careful observation regarding the repetition of some phrases throughout the Abstract, Results, and Conclusion sections. The article has been rewritten some parts, based on your insightful comments.

We are deeply grateful for the constructive feedback provided by the reviewers, which has guided us in refining our manuscript to meet the highest standards of academic rigor and practical applicability. We hope that the revised manuscript meets your expectations. We remain open to any further suggestions to ensure the quality of the final publication.

Sincerely,
